# Comparison of Salivary Stress Biomarkers in Patients with and without Periodontal Diseases: A Cross-sectional Study

**DOI:** 10.3290/j.ohpd.c_2741

**Published:** 2026-06-23

**Authors:** Rana Hameed Jabr, Omar Husham Ali

**Affiliations:** a Rana Hameed Jabr Master’s Student, College of Dentistry, Baghdad University, Baghdad, Iraq. Research concept and design, collected, analyzed, and interpreted the data, wrote, critically revised, and approved the manuscript.; b Omar Husham Ali Assistant Professor, College of Dentistry, Baghdad University, Baghdad, Iraq. Research concept and design, analyzed and interpreted the data, critically revised and approved the manuscript.

**Keywords:** alpha-amylase, chromogranin A, cortisol, periodontitis, stress.

## Abstract

**Purpose:**

This study aimed to evaluate the salivary concentrations of cortisol (Cort), chromogranin A (CgA), and alpha-amylase (sAA) in periodontally healthy individuals and individuals with periodontal disease, and to investigate their association with perceived psychological stress, as measured by the Perceived Stress Scale (PSS).

**Materials and Methods:**

The study included 400 participants aged ≥18 years (117 healthy participants, 140 with gingivitis, and 143 with periodontitis). Unstimulated salivary samples were collected from 90 participants systematically randomized from the 400 enrolled in this study. Subsequently, psychological stress was assessed using the PSS, and periodontal parameters were recorded. The ELISA test was used to measure salivary levels of Cort, CgA, and sAA.

**Results:**

The means of salivary cortisol concentrations were 33.54 (± 3.77 ng/ml) in controls, 65.89 (± 16.41 ng/ml) and 47.06 (± 4.55 ng/ml) in the gingivitis and periodontitis groups, respectively. Mean CgA concentrations were 724.75 (± 90.70 pg/ml) in controls, 1133.47 (± 302.49 pg/ml) in gingivitis, and 1526.30 (± 433.81 pg/ml) in periodontitis. Additionally, the mean of sAA levels was 24.40 (± 6.91 ng/ml) in the control group, 31.44 (± 3.91 ng/ml) and 34.13 (± 5.64 ng/ml) in the gingivitis and periodontitis groups, respectively. PSS scores were considerably higher in individuals with periodontal disease than in healthy controls.

**Conclusion:**

Salivary levels of Cort, CgA, and sAA were elevated in individuals with periodontal disease compared to healthy controls. These biomarkers may serve as supportive indicators in the assessment of periodontal disease. However, further studies are required to validate these findings.

Periodontal diseases are traditionally defined as the progressive deterioration of the hard and soft tissues supporting the teeth, caused by abnormal immune responses and dysbiotic bacterial communities in periodontal tissues.^25^ Periodontitis is a common, multifactorial, chronic inflammatory condition that affects the supporting structures of the teeth and is frequently overlooked by individuals.^1, 23^

Numerous studies involving humans and animals demonstrate that stress influences microbial profiles and colonization, contributing to microbial dysbiosis, disease, and associated immunological alterations.^14^ Psychological stress can alter immune responses and increase susceptibility to periodontal disease by promoting the release of inflammatory mediators.^33^ Acute stressors such as infections and surgery frequently suppress cellular immunity, while the innate immune system is upregulated.^43^ Chronic stress-related disorders can lead to behavioral changes, such as irregular periodontal maintenance visits and poor adherence to periodontal therapy. Additionally, previous studies have shown that the COVID-19 pandemic negatively affected patients’ psychological status and increased their fear of visiting dental clinics.^2, 11^ These factors have long been recognized as contributors to the development of periodontal disease and tooth loss.^15^ Furthermore, neurobiological connections between stress and periodontitis involve activation of the adrenergic nerve signaling axis. This activation may alter vascular function, reduce blood flow, and impair wound healing and immune responses.^39^

Smokers commonly indicate that stress reduction and relaxation are the main reasons for smoking. The bidirectional association between smoking and stress indicates that acute stress exposure in smokers leads to an increase in smoking and cigarette cravings.^9^

Cortisol is one of the most researched hormones linked to stress. The glucocorticoid hormone is produced when psychological stress activates the hypothalamic-pituitary-adrenal (HPA) axis. About 34% of people have temporomandibular joint disorders (TMDs), and up to 18% have bruxism in addition to TMDs.^38^ Cort measurements proved to be a reliable biomarker for assessing psychological stress in relation to TMDs.^40^ Increased cortisol levels lead to a compromised immune response, because it can interfere with cytokine production and inhibit the activity of critical immune cells.^16^ This disruption could lead to decreased T-cell activation and decreased antibody production, which ultimately disrupts the ability of the body to defend against infections and maintain overall health.

Cortisol exposure alters the activity profile of the oral microbiome. It induces an exaggerated response from the host’s immune system, both of which elevate the risk of periodontitis development.^47^ Cortisol can enhance the expression of type IX secretion system (T9SS) associated genes, including mfa5, thereby facilitating surface translocation and potentially increasing the virulence of *Porphyromonas gingivalis*.^24^

Chromogranin A (CgA) is an acidic glycoprotein with a molecular weight of 48 kDa and 439 amino acids; it belongs to the granin family of proteins. The N-terminal CgA fragment vasostatin I (CgA1-76; VS-I) exhibits numerous biological actions, including antibacterial and antifungal properties, and affects vessel tension and vasculature while modifying cell adhesion.^12^ There are limited studies demonstrating an association between psychological health and salivary CgA levels; however, CgA has recently received increased attention for its involvement in stress and associated disorders.^32^ Stress induces the release of CgA, which plays a part in the innate immune system due to its significantly cationic nature.^8^ Elevated salivary CgA levels have been reported in several conditions, such as dry mouth.^45^

Numerous studies on stress have consistently demonstrated elevated salivary alpha-amylase (sAA) levels under stress.^4^ sAA has been shown to influence the formation of dental biofilms and bacterial adherence to tooth surfaces, which may influence the pathogenesis and progression of periodontal diseases.^22^ In-vitro studies indicate that sAA may act as an anti-biofilm agent against biofilm-forming bacterial species, such as *Staphylococcus aureus* and *Pseudomonas aeruginosa*.^27^ In contrast, sAA interacts with the amylase-binding protein of plaque-forming streptococci, thereby initiating biofilm formation.^26^

Saliva contains a variety of biomarkers that may serve as a noninvasive adjunctive biological medium for monitoring periodontal disorders.^6,20^ Furthermore, salivary components, such as exosomes,^17^ may reflect systemic conditions. The process of collecting saliva is simple and rarely requires advanced equipment or trained examiners.

This research was conducted to determine whether the salivary stress biomarkers (Cort, CgA, sAA) provide sufficient accuracy to distinguish between periodontal health and disease. Based on existing evidence, we hypothesized that salivary stress biomarkers (Cort, CgA, and sAA) are elevated in patients with periodontal disease compared to periodontally healthy individuals. Furthermore, we hypothesized that selected salivary stress biomarkers may be associated with disease severity and PSS scores.

## MATERIALS AND METHODS

### Study Design and Settings

In this cross-sectional study, patients were examined who visited the dental clinics at the Department of Periodontics, College of Dentistry, University of Baghdad, Iraq, between November 2024 and May 2025. A total of 400 subjects were recruited for the study, all of whom were systemically healthy. The study was explained verbally to all participants who met the specified inclusion and exclusion criteria, and they were asked to sign an informed consent form. The methodology of the current study adhered to the Declaration of Helsinki and its subsequent updates regarding human research. The Ethics Committee of the University of Baghdad’s College of Dentistry approved the protocol (reference number: 975; project number: 975624; date: 19\11\2024). The study was assessed and confirmed to be compliant with the STROBE (Strengthening the Reporting of Observational Studies in Epidemiology) guidelines.

### Eligibility

The study included participants aged ≥18 years with at least 20 teeth (excluding third molars). Eligible participants were systemically healthy individuals, both male and female, with no history of systemic diseases such as diabetes mellitus or cardiovascular disease.

Exclusion criteria included participants with orthodontic appliances or dental implants, those with recent use of antimicrobials or anti-inflammatory medications, and those using drugs known to affect salivary or serum cortisol levels. Individuals with endocrine disorders (e.g., pituitary or adrenal tumors, Cushing’s syndrome), pregnant or lactating women, and those taking contraceptive pills were also excluded.

Additionally, individuals with a history of alcohol abuse, recent periodontal therapy, or oral lesions not associated with periodontitis (e.g., lichen planus or aphthous ulcers) were excluded from the study.

### Clinical Examination

A single calibrated examiner recorded complete periodontal charting for each participant, including the plaque index (PI), bleeding on probing (BOP), probing pocket depth (PPD), and clinical attachment loss (CAL). Each patient’s mouth was examined using a UNC-15 periodontal probe (MEDSEY 546-1; Maniago, Italy). The presence or absence of plaque was assessed using the full-mouth plaque score (1 and 0),^36^ BOP was recorded as 1 when bleeding occurred within 30 s and 0 when no bleeding was observed within this period. The whole mouth was examined at six sites per tooth, except for plaque scores, which were assessed on four surfaces. The clinical examination excluded third molars. Calibration sessions between the main researcher and a gold-standard periodontics specialist were conducted before the study began for all clinical parameters. Readings were allowed to be taken at least 2 h apart.

### Study Groups

Participants meeting the inclusion criteria were categorized into three groups according to the 2017 World Workshop on the New Classification of Periodontal and Peri-Implant Diseases and Disorders.^10^ All participants, including healthy controls, were recruited from individuals attending the Department of Periodontics for routine check-ups or other non-periodontal complaints, and underwent a comprehensive periodontal examination. Periodontally healthy participants were defined as those with an intact periodontium, probing depths ≤ 3 mm, minimal BOP (<10%), and no history of periodontal disease. Patients in the gingivitis group exhibited BOP ≥ 10%-30% (localized), > 30% (generalized), and PPD ≤3. A patient was diagnosed with periodontitis if any of the following conditions existed: interdental CAL is detectable at ≥2 non-adjacent teeth, or buccal or oral CAL ≥3 mm with pocketing >3 mm is detectable at ≥2 teeth, further classified according to the 2017 World Workshop classification into stages (I–IV) and grades (A–C), based on disease severity and risk of progression.^48^

Each patient’s demographic characteristics were documented, including gender, age, income level, occupation, smoking habits, educational level, and toothbrushing frequency. Concerning smoking habits, current smokers were divided into groups based on how many cigarettes they smoked each day (light: 1 to 9, moderate: 10 to 19, heavy: ≥20). Educational level was classified as (illiterate, primary school, secondary school, or academic). The threshold for those with low incomes (<5.5 dollars per day) was evaluated based on a recently revealed report.^5^ The occupation was categorized as either employed (paid full-time or part-time position) or unemployed (retired or currently unemployed). The Arabic validated version of the PSS^3^ was used to assess perceived stress. The scale consists of 10 items scored on a 5-point Likert scale, with total scores ranging from 0 to 40, where higher scores indicate greater perceived stress. In the primary analysis, PSS scores were treated as a continuous variable. For descriptive purposes only, scores were categorized as low (0–13), moderate (14–26), or high (27–40) perceived stress. These categories were not used as diagnostic cut-offs.

In addition, body mass index (BMI) was calculated as body weight (in kg) divided by height (in meters) squared (kg/m^2^). Waist and height were measured in centimeters. The waist-to-height ratio (WHtR) was calculated as waist divided by height.

### Sample Size

To calculate the sample size, Sharma’s equation^44^ for the cross-sectional study at a 95% confidence interval with a 5% error margin was used:

Sample size = (Z1-α/2)^2^ × (p) × (q) /d^2^

^where Z1-^α/2 = a standard value and a critical value for the equivalent level of confidence, P = expected prevalence, q = 1-p, d = precision or margin of error with Z1-α/2 = 1.96, P = 0.4, and q = 0.6. The calculated sample size was 400 participants. Saliva samples were obtained from patients selected via systematic random sampling; every fifth eligible patient identified during recruitment was included. Following randomization, a subsample of 90 participants was selected for salivary biomarker analysis. The use of a subsample for biomarker analysis was based on both methodological and practical considerations. From a methodological perspective, salivary stress biomarkers exhibit sufficient variability and effect sizes to be reliably detected with moderate sample sizes. From a practical perspective, ELISA-based biochemical assays are resource-intensive, requiring specialized laboratory procedures, controlled experimental conditions, and high-cost reagents. This approach is consistent with previous cross-sectional studies investigating stress-related biomarkers in periodontal disease, in which moderate sample sizes have been sufficient to detect meaningful differences in biomarker levels.^41^ Therefore, the biomarker analysis was considered exploratory and hypothesis-generating, as it was conducted on a subsample not powered for definitive conclusions.

Whole unstimulated saliva was collected passively by drooling; to minimize circadian rhythm changes between patients, saliva samples were collected between 9:00 and 11:00 a.m. An ELISA kit (Elabscience Biotechnology; Wuhan, Hubei Province, China) was used to quantitatively determine salivary stress biomarker levels (Cort, CgA, and sAA) according to the manufacturer’s instructions.

### Statistical Analysis

Statistical analysis of the data, including both descriptive and inferential statistics, was conducted using SPSS version 27.0 (IBM; Armonk, NY, USA). The Shapiro-Wilk test was performed to assess the normality of the data. The descriptive data were presented as mean ± standard deviation (SD). A p-value less than 0.05 was considered statistically significant. The chi-squared test was employed to compare categorical variables across the study groups, including sex distribution, smoking status, educational level, employment status, income levels, BMI categories, and WHtR categories. A one-way ANOVA was conducted to evaluate differences in continuous variables, including age, sleep duration, brushing frequency, clinical periodontal parameters, and salivary biomarker levels (Cort, CgA, sAA) across the healthy, gingivitis, and periodontitis groups. Post-hoc analyses were performed using Tukey’s test for continuous variables and Bonferroni-adjusted pairwise comparisons for categorical variables. Receiver Operating Characteristic (ROC) curve analysis was used to evaluate the preliminary diagnostic accuracy of salivary Cort, CgA, and sAA, providing estimates of the area under the curve (AUC), sensitivity, specificity, and optimal cut-off points. Multiple regression analysis was performed to evaluate the independent associations between salivary stress biomarkers and periodontal disease parameters (BOP, CAL), while controlling for potential confounders. The Variance Inflation Factor (VIF) was used to assess multicollinearity among independent variables in the regression model. Effect sizes (Cohen’s d) were calculated to assess the magnitude of differences between groups across pairwise comparisons.

## RESULTS 

A total of 460 participants were initially assessed for eligibility. A total of 60 individuals were excluded based on the study’s exclusion criteria, resulting in 400 participants being enrolled. The study population was divided into three groups: healthy control (n = 117), gingivitis (n = 140), and periodontitis (n = 143). Of these 400 participants, a subset of 90 individuals was selected for saliva sampling (27 healthy controls, 30 patients with gingivitis, and 33 patients with periodontitis). Salivary samples were collected before clinical examination, and all participants underwent a comprehensive periodontal assessment.

### Demographic Data of the Study Populations

The demographic and anthropometric characteristics of the study groups are presented in Table 1. Statistically significant differences were observed in sex distribution, age, smoking status, educational level, BMI, WHtR, and brushing frequency (p < 0.05).

**Table 1:** Demographic data of the study populations (n= 400)

Variable	Control	Gingivitis	Periodontitis	p-value
**Sex**				
Male n (%)	47 (40.2)^a^	78 (55.7)^b^	78 (54.5)^b^	0.021*
Female n (%)	70 (59.8)^a^	62 (44.3)^b^	65 (45.5)^b^	
**Age**				
Mean ± SD	27.94±8.443^a^	29.74±9.915^a^	45.73±12.83^b^	0.001*
**Smoking**				
Non-smoker	104 (88.9)^a^	89 (63.6)^b^	108 (75.5)^c^	0.001*
Smoker	13 (11.1)^a^	51 (36.4)^b^	35 (24.5)^c^	
**Smoking habit**				
Low	11(9.4)^a^	3 (2.1)^b^	2 (1.4)^b^	0.001*
Moderate	2 (1.7)^a^	8 (5.7)^b^	1 (0.7)^a^	
Heavy	0^a^	40 (28.6)^b^	32 (22.4)^b^	
**Educational level**				
Illiterate	0^a^	1 (0.7)^a^	11 (7.7)^b^	0.001*
Primary	7 (6)^a^	32 (22.9)^b^	34 (23.8)^b^	
Secondary	17 (14.5)^a^	65 (46.4)^b^	59 (41.3)^b^	
Academic	93 (79.5)^a^	42 (30)^b^	39 (27.3)^b^	
**Employment status**				
Retired	1 (0.9)^a^	0^a^	12 (8.4)^b^	0.001*
Employed	58 (49.6)^a^	68 (48.6)^a^	71 (49.7)^a^	
Unemployed	58 (49.6)^a^	72 (51.4)^a^	60 (42)^b^	
**Income level**				
Low	61 (52.1)	74 (52.9)	59 (41.3)	0.152
Middle	56 (47.9)	66 (47.1)	84 (58.7)	
**BMI**				
Normal	67 (57.3)^a^	55 (39.3)^b^	52 (36.4)^b^	0.046 *
Overweight	39 (33.33)^a^	47 (33.6)^a^	56 (39.2)^a^	
Obese	11 (9.4)^a^	38 ( 27.1)^b^	35 (24.5)^b^	
**WHtR**				
Low risk	44 (37.6)^a^	39 (27.9)^a^	23 (16.1)^b^	0.001*
High risk	73(62.4)^a^	101 (72.1)^a^	120 (83.9)^b^	
**Sleep hours**				
Mean ±SD	6.94±1.391	6.86±1.838	6.52±1.76	0.094
**Brushing frequency**				
Mean ±SD	2.27±0.624^a^	0.68±0.651^b^	0.49±0.55^b^	0.001*
* Statistically significant using the chi-squared test; one-way ANOVA. BMI: body mass index; WHtR: waist-to-height ratio. Different superscript letters indicate statistically significant pairwise differences between groups. Groups with the same supercript letter are not statistically significantly different.				

### The Perceived Stress Questionnaire

Table 2 shows the distribution of participants by stress level throughout the study groups. Mean PSS scores differed statistically significantly among the study groups (p < 0.001).

**Table 2:** The distribution of participants based on stress in each group (n = 400)

Stress level	Healthy (n = 117)	Gingivitis (n = 140)	Periodontitis (n = 143)
Low (0–13)	33 (28.2%)	9 (6.4%)	13 (9.1%)
Moderate (14–26)	70 (59.8%)	99 (70.7%)	87 (60.8%)
High (27–40)	14 (12.0%)	32 (22.9%)	43 (30.1%)
Mean ± SD	18.30 ± 6.10	21.74 ± 5.67	23.13 ± 5.75
p < 0.001* (ANOVA)			
Mean differences between groups were analyzed using one-way ANOVA.			

### Clinical Parameters Among Study Groups

Statistical analysis revealed significant differences among the three groups across all measured clinical parameters (p < 0.05) (Table 3). The majority of periodontitis cases were classified as Stage III (64.3%) and Grade C (74.1%), indicating that most patients had moderate to severe disease with a high rate of progression.

**Table 3:** Descriptive and statistical analysis of clinical periodontal parameters among the study groups (n = 400)

Clinical parameters	Groups	Mean	±SD	Min	Max	p-value*
Staging and grading, n (%)						
PI	Control	24.16	7.524	12	42	0.001*
	Gingivitis	67.10	16.12	30	94	
	Periodontitis	70.87	13.74	36	96	
BOP	Control	5.94	1.31	4	9	0.001*
	Gingivitis	47.37	15.77	16	88	
	Periodontitis	51.74	14.90	22	92	
PPD	Gingivitis	2.52	0.32	1.40	3.20	0.001*
	Periodontitis	5.10	0.66	4	6.80	
CAL	Periodontitis	4.96	1.44	2.89	6.38	
Stage I	0					
Stage II	28 (19.6)					
Stage III	92 (64.3)					
Stage IV	23 (16.1)					
Grade A	1 (0.7)					
Grade B	36 (25.2)					
Grade C	106 (74.1)					
PI: plaque index; BOP: bleeding on probing; CAL: clinical attachment loss; Min: minimum; Max: maximum. *Statistically significant (one-way ANOVA) at p ≤ 0.05.						

### Salivary Levels of Cort, CgA, and sAA

**Fig 1:**
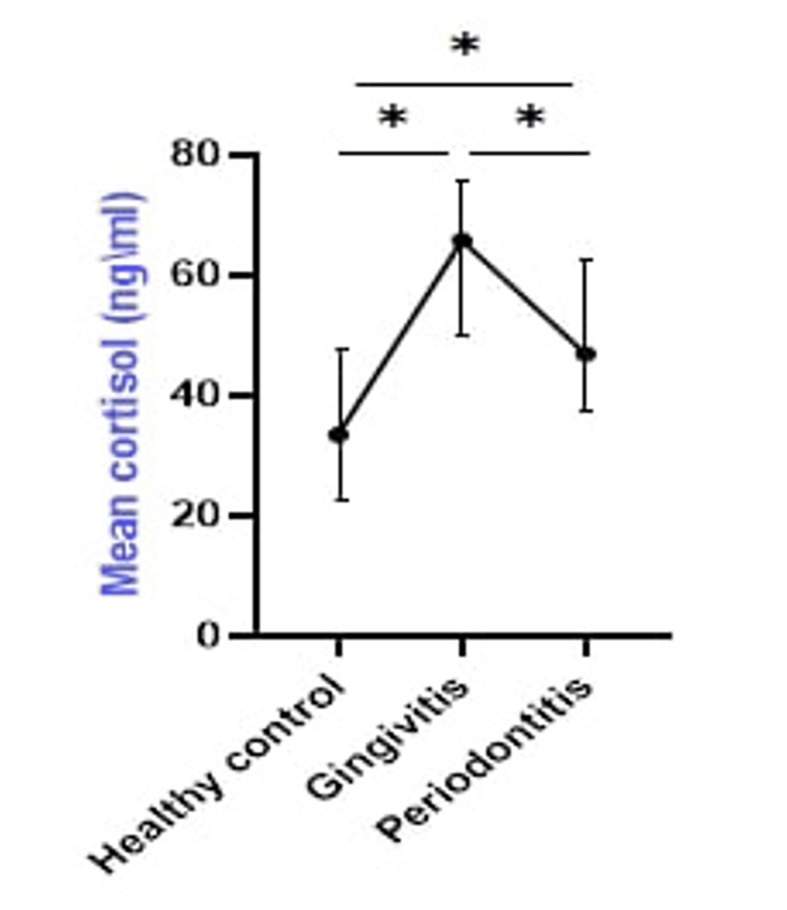

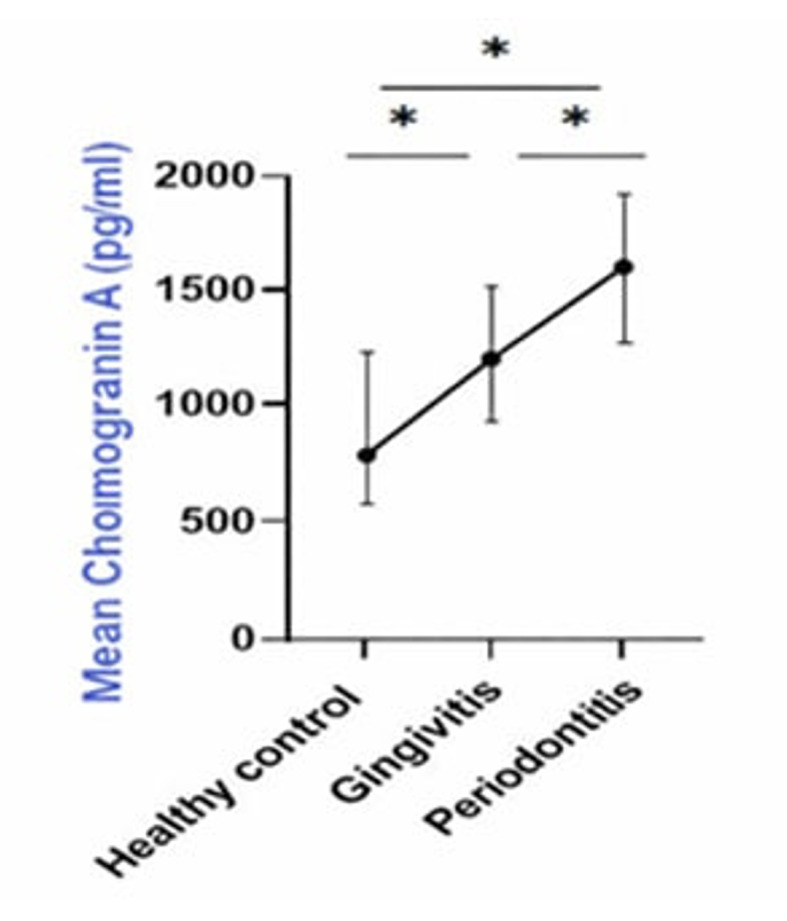

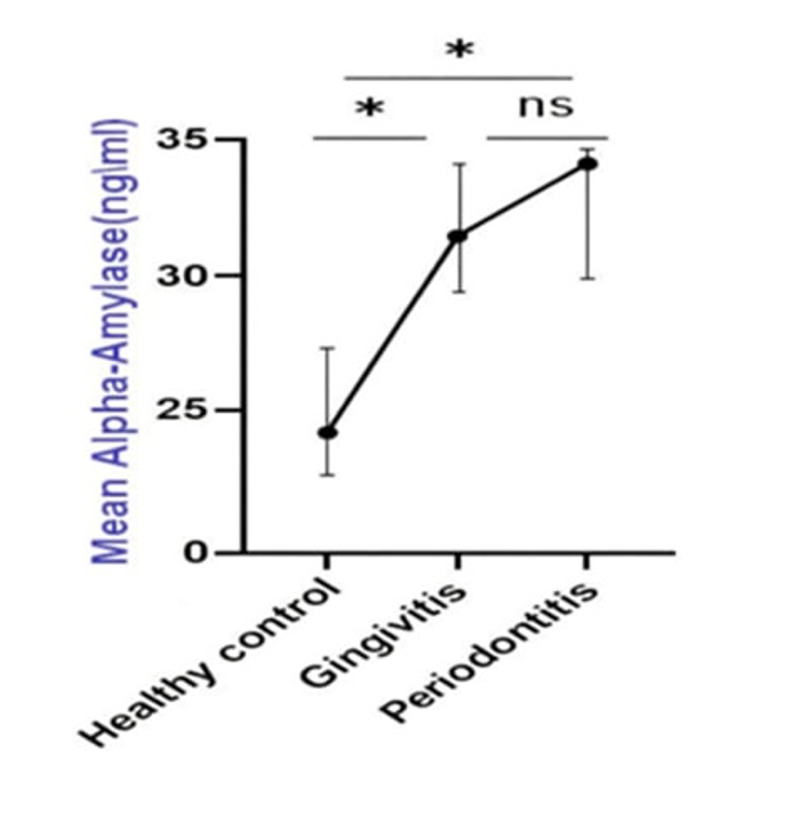
Comparison of mean salivary cortisol, chromogranin A, and alpha-amylase levels among study groups. ns: non-significant; *statistically significant.

The salivary levels of stress biomarkers are shown in Table 4. The data show Cort, CgA, and sAA concentrations differed statistically significantly between the control, gingivitis, and periodontitis groups (P = 0.001). Large effect sizes were observed across all biomarkers (Cohen’s d ranging from 0.55 to 3.21), indicating substantial between-group differences. Additionally, the comparison of mean salivary Cort, CgA, and sAA levels among the healthy controls, gingivitis, and periodontitis groups is shown in Fig 1.

**Table 4:** Descriptive and statistical tests of biomarkers for the study groups (n= 90)

Biomarker	Groups	Mean	±SD	Min	Max	p-value
Cort (ng/ml)	Control	33.54	3.77	23.851	37.48	0.001*
	Gingivitis	65.89	16.41	39.784	91.858	
	Periodontitis	47.06	4.55	41.601	69.842	
sAA (ng/ml)	Control	24.40	6.91	5.413	32.317	0.001*
	Gingivitis	31.44	3.91	26.861	45.000	
	Periodontitis	34.13	5.64	21.150	46.77	
CgA (pg/ml)	Control	724.75	90.70	586.044	956.493	0.001*
	Gingivitis	1133.47	302.49	902.709	2079.314	
	Periodontitis	1526.30	433.81	1179.31	2585.047	
Cortisol: Cort; alpha-amylase: sAA; chromogranin A: CgA. *Statistically significant at p ≤ 0.05 (one-way ANOVA).						

### Diagnostic Accuracy

**Fig 2:**
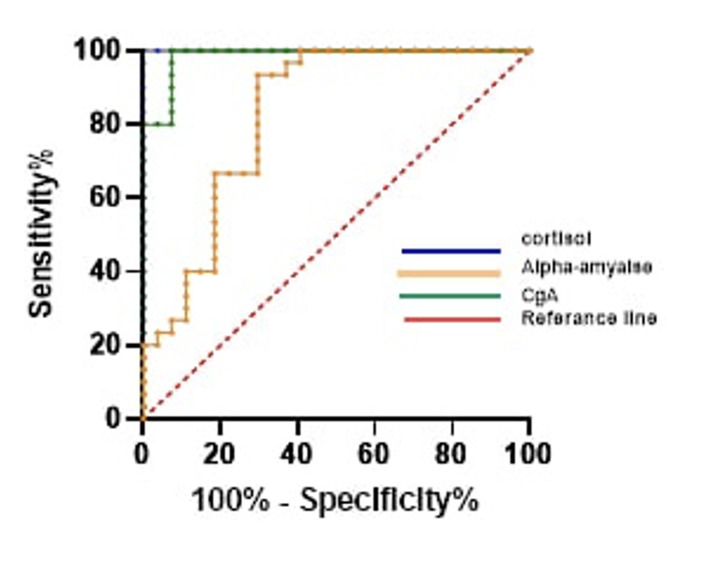

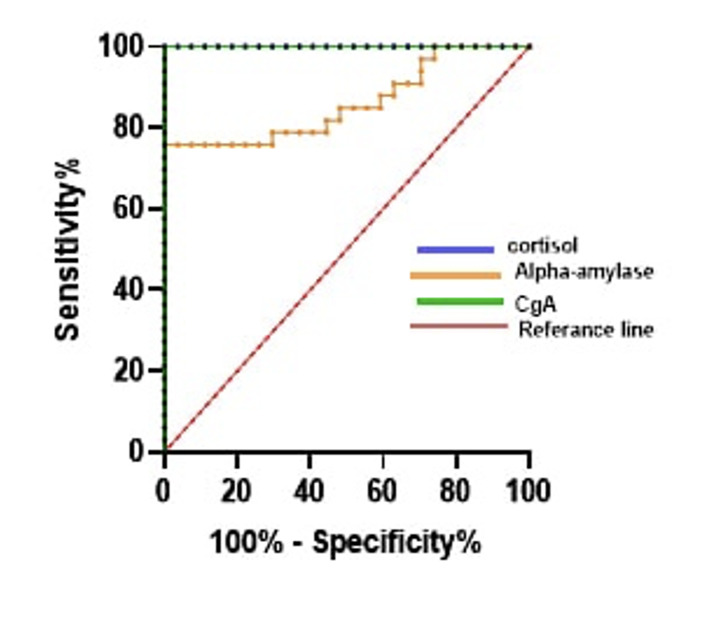

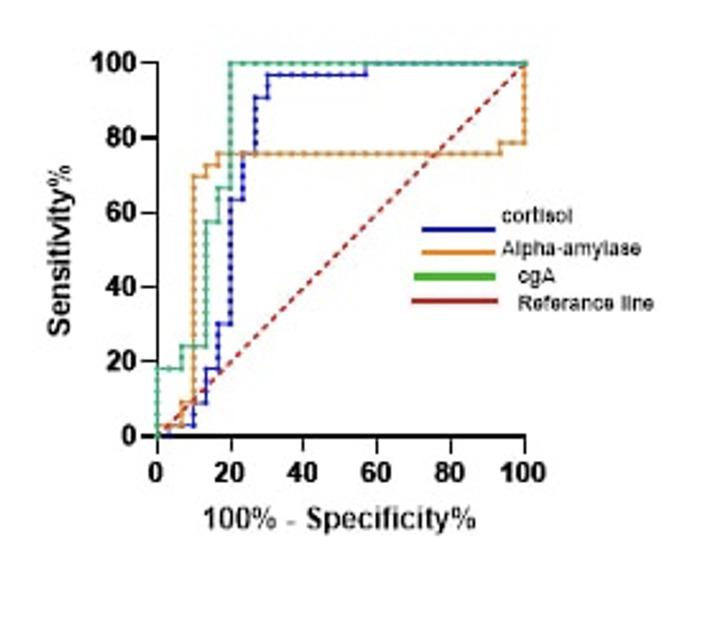
ROC curves for Cort, CgA, and sAA: (a) control vs gingivitis; (b) control vs periodontitis: (c) gingivitis vs periodontitis.

The diagnostic accuracy of salivary stress biomarkers for distinguishing periodontal health from disease was assessed using ROC analysis, as shown in Table 5 and Fig 2. Cortisol and CgA demonstrated preliminary diagnostic accuracy, particularly in distinguishing healthy controls from diseased groups. However, their discriminatory capacity was reduced when differentiating between gingivitis and periodontitis. sAA exhibited overall moderate-to-low diagnostic performance across comparisons, with the lowest discriminatory ability in distinguishing gingivitis from periodontitis.

**Table 5:** Sensitivity, specificity, and cut-off point of biomarkers (n= 90)

Groups	Test result variable(s)	AUC		p-value	Optimal cutoff point	% Sensitivity	% Specificity
Control vs gingivitis	Cort	1.000	excellent	<0.0001	> 37.44	100.0	100.0
	sAA	0.8272	good	<0.0001	> 27.00	96.67	62.96
	CgA	0.9852	excellent	<0.0001	> 973.4	66.67	100.0
Control vs periodontitis	Cort	1.000	excellent	<0.0001	> 39.54	100.0	100.0
	sAA	0.8608	good	<0.0001	> 34.78	69.70	100.0
	CgA	1.000	excellent	<0.0001	> 1170	96.97	100.0
Gingivitis vs periodontitis	Cort	0.7899	air	<0.0001	< 60.24	96.97	63.33
	sAA	0.6859	poor	0.0113	> 34.81	69.70	90.00
	CgA	0.8697	good	<0.0001	> 1170	96.97	80.00
AUC: area under the curve; Cort: cortisol; sAA: alpha-amylase; CgA: chromogranin A.							

### Multiple Linear Regression

Table 6 presents the outcomes of the regression models. Variables included in the regression models (age, smoking, BMI, and brushing frequency) were selected based on their clinical relevance and statistical significance in preliminary analyses to minimize the risk of overfitting. In Model 1, both the gingivitis and periodontitis groups showed statistically significantly higher levels of all salivary biomarkers compared with the healthy group (p < 0.05). In Model 2, BOP and CAL were statistically significantly associated with salivary Cort and CgA (p < 0.05). However, their associations with sAA were not statistically significant. Age remained non-significant across all biomarkers, with no evidence of multicollinearity (VIF < 5).

**Table 6:** Multiple linear regression analysis of salivary stress biomarkers according to demographic, behavioral, and clinical periodontal variables (n = 90)

Model I	Cort R² = 0.48 (B ± SE) (ng/ml)	p-value		sAA R² = 0.36 (B ± SE) (ng/ml)	p-value		CgA R² = 0.54 (B ± SE) (pg/ml)	p-value	
Model II	Cort R² = 0.42 (B ± SE) (ng/ml)	p-value	VIF	sAA R² = 0.36 (B ± SE) (ng/ml)	p-value	VIF	CgA R² = 0.51 (B ± SE) (pg/ml)	p-value	VIF
Age	0.04 ± 0.06	0.45		0.05 ± 0.10	0.53		5.00 ± 6.20	0.43	
Gingivitis	17.90 ± 4.10	<0.001*		5.60 ± 2.00	0.008*		370 ± 92	0.008*	
Periodontitis	11.20 ± 3.80	0.004*		7.00 ± 2.20	0.002*		710 ± 105	<0.001*	
Smoking	2.80 ± 1.10	0.014*		1.30 ± 0.95	0.17		235 ± 82	0.005*	
Brushing frequency	-2.05 ± 0.80	0.012*		-2.70 ± 1.10	0.018*		-150 ± 58	0.011*	
BMI	0.90 ± 0.39	0.026*		0.58 ± 0.48	0.23		84 ± 34	0.019*	
Age	0.04 ± 0.05	0.46	1.7	0.05 ± 0.09	0.56	1.6	5.0 ± 6.0	0.44	1.8
BOP	0.044 ± 0.015	0.005*	2.6	0.065 ± 0.022	0.07	2.4	5.0 ± 1.9	0.010*	2.5
CAL	1.48 ± 0.43	0.001*	2.9	2.20 ± 0.85	0.06	2.7	135 ± 52	0.012*	2.8
R²: coefficient of determination; B = regression coefficient; SE = standard error; VIF: variance inflation factor. In Model I, age, periodontal group (healthy as reference, gingivitis, periodontitis), smoking status, BMI, and brushing frequency were included as independent variables. In Model II, age and clinical periodontal parameters BOP and CAL were included to assess the effect of periodontal inflammation and disease severity. *Statistically significant at p ≤ 0.05.									

## DISCUSSION

The present findings of this study suggest that stress-related responses may be associated with the development and progression of periodontal diseases, as salivary stress biomarkers (Cort, CgA, and sAA) are statistically significantly elevated in individuals with periodontal disease compared with healthy controls. When an individual is under stress, corticotropin-releasing hormone (CRH) can stimulate gingival mast cells. Periodontitis may result from the mast cells’ subsequent release of pro-inflammatory molecules, additional neuropeptides, and cytokines.^37^

This finding supports the role of stress-related hormonal dysregulation in periodontal inflammation and is consistent with previous studies reporting elevated cortisol levels in periodontal diseases.^13,21^ Notably, our study found that cortisol levels were higher in the gingivitis group than in the periodontitis group. This finding may be attributed to the inflammatory nature of gingivitis, which is characterized by increased levels of pro-inflammatory cytokines, both locally and systemically, including IL-6 and TNF-α. These cytokines are linked to elevated levels of both acute and chronic stress.^42^ In response to inflammatory and psychological stressors, these cytokines are closely associated with activation of the HPA axis, which increases cortisol release.

The present study’s findings demonstrated that the average salivary CgA level was markedly lower in the control group than in the gingivitis and periodontitis groups, with the highest mean value observed in the periodontitis group (p = 0.001). This pattern may indicate an association between CgA levels and disease severity, suggesting a potential link to periodontal inflammation, consistent with previous studies.^18,29^ CgA showed potential as a supportive marker not only in differentiating diseased individuals from healthy individuals but also in distinguishing gingivitis from periodontitis, suggesting its sensitivity to disease progression and severity. This finding is essential because it highlights CgA as a potentially sensitive biomarker for staging periodontal disease. This is consistent with a previous study,^31^ which reported decreased levels of this neuroendocrine marker (CgA) after nonsurgical periodontal therapy, further confirming the association between elevated CgA levels and inflammation in the course of periodontal disorders. However, the ROC analysis results need to be carefully assessed. As an exploratory analysis, the ROC results may be subject to overfitting; therefore, before any diagnostic determinations can be made, external validation in larger, independent cohorts is required. On the other hand, a previous study ^13^ reported that CgA levels were not statistically significantly correlated with the severity of chronic periodontitis.

Among the clinical periodontal parameters, multiple regression analysis demonstrated that both CAL and BOP were independently and positively associated with salivary Cort and CgA. The final regression models incorporated CAL and BOP, rather than PPD, which may be affected by gingival swelling or the formation of pseudo-pockets. CAL is a more precise indicator of the true severity of the condition, thereby reflecting cumulative and irreversible periodontal damage. Similarly, although PI primarily indicates plaque buildup rather than the host’s inflammatory response, BOP reflects an active inflammatory condition. These findings indicate that elevated biomarker levels reflect underlying biological and inflammatory processes associated with periodontal disease.

This study revealed that the concentration of sAA was markedly reduced in the control group relative to the gingivitis and periodontitis groups, with the periodontitis group exhibiting the highest mean concentration. The elevated levels of sAA observed with increased severity of periodontal disease may be explained by its association with sympathetic nervous system activation. Periodontal inflammation can act as a physiological stressor, triggering the sympathetic response and increasing sAA secretion. This increase may represent a part of the body’s adaptive or protective mechanism aimed at counteracting the progression of inflammatory processes within the oral cavity. The current findings corroborate those of earlier studies,^19, 35^ which likewise observed elevated sAA concentrations in patients with periodontal disease. Furthermore, the observed decrease in sAA levels following periodontal therapy may reflect reduced inflammation and improved periodontal health.^34^ However, in this study, sAA showed a limited ability to differentiate between gingivitis and periodontitis and did not exhibit a statistically significant association with clinical periodontal parameters as indicated by multiple regression analysis. These results suggest that while sAA can serve as a supportive indicator for identifying periodontal disease, it is more effective when combined with other biomarkers than when used alone. Its role appears to be more related to general stress-related responses than to the specific severity of periodontal tissue destruction.

Regarding demographic data, there was a noticeable age difference between the groups. The group with periodontitis had the highest mean age, while the healthy controls had the lowest. Older individuals are more susceptible to certain inflammatory, infectious, and autoimmune conditions, including periodontal disorders. Periodontal disease and bone loss tend to worsen with age, possibly due to prolonged exposure to dental plaque, reflecting the patient’s oral history. Age was considered a potential confounding factor and was adjusted for in the multiple regression models along with other relevant variables, particularly smoking status. The associations between periodontal disease status and salivary biomarkers remained statistically significant after adjusting for age (Table 6, Model I), indicating that these elevations are independent of age differences between groups.

The present findings demonstrated positive associations between BMI, WHtR, and periodontal disease (p ≤ 0.05). This is supported by other studies that identify obesity as a risk factor for periodontal diseases^7,28^ and caries.^46^ These studies suggest that hormonal and metabolic disturbances are likely to play an essential role in modulating the host inflammatory response and disrupting immune homeostasis, thereby contributing to the progression and severity of periodontal tissue destruction. Obesity is characterized by a dysregulated HPA axis, modified cortisol production patterns, and abnormal diurnal salivary cortisol rhythms.^30^

A progressive increase in perceived stress across the study groups was found in this study. This pattern suggests a potential association between higher perceived stress and worsening periodontal condition. It is important to note that PSS was used to assess perceived stress rather than as a diagnostic tool, and categorical groupings were used for descriptive purposes only.

The main strength of the present study was the incorporation of stress evaluation using the PSS in conjunction with measurement of salivary stress biomarker levels. The integration of subjective and objective methodologies facilitated a more accurate, non-invasive assessment of the potential association between stress and periodontal disease, thereby enhancing the clinical relevance of the findings.

The findings of this study may have limited generalizability. A key limitation is that salivary biomarker analysis was conducted on a relatively small subsample, which may have reduced statistical power. Therefore, the biomarker findings should be interpreted as exploratory and hypothesis-generating rather than confirmatory. Although large effect sizes (Cohen’s d > 0.5) suggest meaningful between-group differences, smaller effects may not have been detected. In addition, the cross-sectional design precludes causal inference, and residual confounding cannot be entirely excluded despite adjustment for several variables. Furthermore, the study lacked objective stress assessments performed by trained professionals. Future longitudinal studies with larger, adequately powered samples are required to validate these findings.

## CONCLUSION

Cort, CgA, and sAA salivary concentrations were markedly elevated in the gingivitis and periodontitis groups compared to the healthy group. Additionally, CgA levels increased with disease severity and showed potential sensitivity and specificity in differentiating gingivitis from periodontitis, although further validation is required. These results suggest that these biomarkers may serve as supportive indicators in the assessment and monitoring of periodontal conditions. Stress levels, as measured by the PSS, were notably higher in disease groups than in the healthy control group.
